# Factors Influencing the Choice Between Multi-Target Stool DNA and Colonoscopy for Colorectal Cancer Screening Among Alaska Native Peoples

**DOI:** 10.3390/life15010120

**Published:** 2025-01-17

**Authors:** Lauren A. Jeffries, Christie A. Flanagan, Lila J. Finney Rutten, John B. Kisiel, Diana G. Redwood

**Affiliations:** 1 Alaska Native Tribal Health Consortium, 3900 Ambassador Dr., Anchorage, AK 99508, USA; 2 Mayo Clinic, 200 1st St SW, Rochester, MN 55905, USAkisiel.john@mayo.edu (J.B.K.)

**Keywords:** Alaska Native, cancer screening, colorectal cancer, health care, screening barriers, colorectal neoplasms/prevention and control, early detection of cancer, surveys and questionnaires

## Abstract

The Alaska Tribal Health System is increasing colorectal cancer (CRC) screening among Alaska Native (AN) peoples, who experience the highest CRC rate in the world. Through a clinical trial (NCT04336397), AN people living in rural/remote Alaska were offered either the previously unavailable multi-target stool DNA test (mt-sDNA; Cologuard^®^) or colonoscopy. From April 2022 to August 2024, 113 (59%) people who completed mt-sDNA testing and 51 (39%) who completed a colonoscopy procedure participated in a survey on factors influencing their screening test choice. The majority of mt-sDNA participants (79%) were aware of the colonoscopy option, while most colonoscopy participants (72%) reported being unaware of the mt-sDNA option. Key mt-sDNA preference themes included not having to travel, less time commitment, childcare and work responsibilities, physical limitations, greater convenience, and the test being less invasive and dangerous than a colonoscopy. In contrast, colonoscopy preference themes included wanting to adhere to screening recommendations, be proactive about preventative health, family history of cancer, awareness of the higher CRC risk among AN peoples, as well as the belief that colonoscopy is more reliable and accurate since it is performed by medical providers. These findings underscore the complex factors influencing CRC screening preferences among rural and remote AN people. Limited access to medical facilities, financial burdens, and personal commitments, along with personal factors, have a substantial bearing on the screening decision-making process. Recognizing and addressing these multifaceted influences can help CRC screening programs better meet community member needs and preferences, ultimately improving screening rates and colorectal health outcomes.

## 1. Introduction

The Alaska Tribal Health System is working to improve cancer prevention and control among Alaska Native peoples, who have the highest rates of colorectal cancer (CRC) in the world [1]. In the United States, American Indian/Alaska Native people have higher incidence (48.6 vs. 35.7 per 100,000 people) and mortality (18.6 vs. 13.1 per 100,000 people) rates than US Whites [2]. For Alaska Native individuals specifically, the rates are even higher. In 2018, the incidence rate among Alaska Native people was 61.9 per 100,000 people, marking the highest recorded rate globally [1]. CRC screening reduces mortality [3], and while Alaska Native CRC screening rates have been rising, only 59% of Alaska Native peoples are up-to-date [4], with screening rates consistently below the national average (67%) [5], reinforcing the need for new screening options for the Alaska Native population.

The state of Alaska spans over 660,000 square miles, making it the largest state by area in the United States [6]. Over half of rural Alaska Native communities are located off the road system and are only accessible year-round by small aircraft. This limits access to CRC screening methods, such as colonoscopy, which require a flight to a regional hub hospital or the Alaska Native Medical Center in Anchorage, Alaska. While the colonoscopy procedure itself is a covered health service for Alaska Native beneficiaries, the cost of travel, including airfare and lodging, is often the patients’ responsibility. This adds a financial burden, in addition to the time that people must be away from their home community as they prepare for the procedure and then fly home afterward. On the system side, flight cancellations due to weather and the limited availability of qualified staff for endoscopy clinics also play a role in procedure availability and accessibility.

Due to logistical challenges surrounding completing a colonoscopy in rural/remote Alaska, at-home screening tests such as the fecal immunochemical test (FIT) are a potentially more accessible screening option. However, the high rates of precancerous polyps and CRC in the Alaska Native population [1,7] have led to colonoscopy being the primary CRC screening test recommended for Alaska Native peoples by the Alaska Native Medical Center [8], with other tests, such as guaiac-based fecal occult blood testing (gFOBT), not being recommended due to false positives associated with a high prevalence of Helicobacter pylori infection-related gastrointestinal bleeding [9,10,11].

In 2014, the multi-target stool DNA test (mt-sDNA; Cologuard^®^, Exact Sciences) was approved for U.S. commercial use. The mt-sDNA screening method has higher sensitivity than other home collection stool options, provides detection of both left- and right-side colon cancers, and offers a unique feature of utilizing an integrated patient navigation and reminder system [12,13,14]. The test is recommended to be completed every three years if the results are normal, and while an abnormal mt-sDNA result still requires a follow-up colonoscopy, early studies have shown that mt-sDNA use may actually increase the yield and quality of follow-up colonoscopies [12,13]. Although mt-sDNA is nationally available, it had not been used in rural and remote Alaska Native communities prior to this study.

As part of a randomized control clinical trial (NCT04336397), Alaska Native people living in one rural/remote region in southwest Alaska were offered either no-cost colonoscopies or mt-sDNA tests for CRC screening [15]. The U.S. Department of Agriculture Economic Research Service classifies this region as frontier and remote, which is characterized by a combination of low population size and high geographic remoteness [16]. To better understand Alaska Native experiences and reasons for choosing either mt-sDNA or colonoscopy for screening, as well as the factors influencing test choice and future screening test preference, we conducted an open-ended telephone survey after screening test completion.

## 2. Materials and Methods

The study was approved by the Alaska Area Institutional Review Board (IRB #2019-04-038) and tribal research oversight committees at the Alaska Native Tribal Health Consortium and the Yukon-Kuskokwim Health Corporation, who also approved this manuscript and presentation of findings. The full intervention study, of which this is a part, is registered at clinicaltrials.gov (NCT04336397).

### 2.1. Eligibility

The Alaska mt-sDNA intervention study methods have been described in detail elsewhere [15]. Briefly, eligible participants were Alaska Native adults aged 45–75 who were eligible to receive health care through the Alaska Tribal Health System, had at least one visit in the previous three years, resided in one of thirty-two intervention communities, and had contact information on file in their medical record. People were excluded from recruitment if they were already adherent to screening guidelines by having a colonoscopy within the past 10 years, a flexible sigmoidoscopy within 5 years, or a fecal occult blood test (FOBT) within 12 months. People were also excluded if they had a history of familial adenomatous polyposis, hereditary non-polyposis CRC, previous colonoscopic evidence of inflammatory bowel disease (including Crohn’s disease), colorectal adenomas, CRC, a first-degree relative diagnosed with CRC at age 60 or younger, or positive FOBT in the last 6 months, as these conditions made them ineligible for mt-sDNA use.

Communities in the participating region were randomly assigned to either intervention or control groups. Intervention communities were further divided into high or medium intervention-intensity arms. In high-intensity intervention communities, a patient navigator based at the regional hospital contacted people by telephone informing them that they were due for CRC screening and offering either colonoscopy or mt-sDNA for screening. People in medium-intensity intervention communities received a mailed outreach letter and health information flyer about the colonoscopy and mt-sDNA screening options. Regardless of the intervention arm or test selected, all people in the intervention received the same patient navigation and assistance to complete their chosen screening test.

### 2.2. Data Collection

From April 2022 to August 2024, people who chose colonoscopy for screening and completed the procedure, or who chose mt-sDNA and had a valid test result, were called and invited to complete a 5 min follow-up survey to elicit information on test preferences and the factors influencing screening choice, as well as thoughts on mt-sDNA as a new screening option in the region. The survey was developed in collaboration with regional tribal health organization partners. For people completing mt-sDNA, the survey included three questions: (1) Why did you choose to use the at-home mt-sDNA test for colon cancer screening?; (2) Were you aware you could have had a colonoscopy for colon cancer screening?; and (3) Why did you choose the mt-sDNA test for colon cancer screening instead of colonoscopy? For people completing colonoscopy, the survey included four questions: (1) Why did you choose to have a colonoscopy for colon cancer screening?; (2) Were you aware of the mt-sDNA take home colon cancer screening test?; (3) Why did you choose a colonoscopy for colon cancer screening instead of mt-sDNA?; and (4) What might make you interested in the mt-sDNA test in the future?

The survey was administered by two research assistants who were not involved in the intervention trial outreach. People were contacted up to two times to complete the survey. Survey responses were transcribed verbatim using Research Electronic Data Capture (REDCap version number 14.0.8), a Health Insurance Portability and Accountability Act compliant data collection software [17,18]. Dedoose software (version number 9.2.5) was used to thematically analyze responses using an inductive qualitative methodology [19,20]. The lead coder created a codebook which was reviewed by a study research assistant. An Inter-Rater Reliability Test was conducted with two independent coders on a 10% sub-sample. Acceptable reliability and consistency in applying codes to excerpts was demonstrated with a Cohen’s kappa statistic of 0.75 for the colonoscopy survey and 0.91 for the mt-sDNA survey [21].

Summary statistics provide an overview of the demographic characteristics of survey respondents. Chi-square tests for proportion differences were used for demographic differences between mt-sDNA survey responders and non-responders, as well as colonoscopy survey responders and non-responders; *p*-values <0.05 were considered to be statistically significant.

## 3. Results

### 3.1. MT-sDNA

A total of 192 Alaska Native people completed a valid mt-sDNA test. Of these, 59% (n = 113) completed the post-screening survey; 55% were male, and 45% were female (Table 1). The majority of respondents were aged 45–60 years (69%), while the remainder were aged 61–75 years (31%). About one-third (39%) were from medium-intensity intervention communities and two-thirds (61%) were from high-intensity intervention communities. There were no significant intervention arm, sex, or age demographic differences between mt-sDNA survey respondents and non-respondents (*p* < 0.05). Among those surveyed, 24% had an abnormal mt-sDNA result, and 76% had a normal result, which was similar to the overall study population.

The survey results (Table 2) revealed consistent themes in the preference for mt-sDNA as a screening option, primarily the perceived ease and convenience of mt-sDNA. Alaska Native respondents found mt-sDNA more convenient (especially in rural communities without road access to a hospital) and appreciated it as a less invasive option for CRC screening. Logistical concerns about completing a colonoscopy also influenced the choice of mt-sDNA. Many participants reported work commitments and childcare responsibilities that made it difficult to take multiple days off for colonoscopy travel required from rural/remote Alaska Native communities.

Negative perceptions of colonoscopy, including fear, embarrassment, and potential discomfort, were also noted factors impacting the screening test decision. Safety concerns were also reported, with participants worried about physical harm from the endoscope and awareness of fellow community members having prior negative experiences from colonoscopy. Health care access challenges included financial concerns related to travel costs, accommodations, and the requirement of a medical escort for colonoscopy, as well as the COVID-19 pandemic that was occurring during the intervention trial. However, people reported specific challenges using mt-sDNA, including issues collecting the sample and weather delaying the sample being able to get to the lab for processing within the test validity window.

### 3.2. Colonoscopy

Of the 130 people who completed a colonoscopy during the intervention, 51 (39%) participated in the post-intervention survey; 39% were male, and 61% were female (Table 1). The majority of respondents were aged 45–60 years (78%), with the remainder aged 61–75 years (22%). A third (39%) were from medium-intensity intervention communities, while 61% were from high-intensity intervention communities. Colonoscopy survey respondents and non-respondents did not differ significantly by intervention arm, sex, or age (*p* < 0.05). Among those who participated in the survey, 39% had normal/hyperplastic polyps, 28% had low risk adenomas, 31% had advanced adenomas (includes adenomas ≥ 1 cm and those containing tubulo-villous or villous histology or high grade dysplasia), and 2% had CRC detected on colonoscopy.

Five key themes emerged from the colonoscopy survey data (Table 2). The most common theme for choosing colonoscopy was that screening had been recommended through outreach phone calls and letters, followed by the desire to pursue preventive health behaviors, and wanting to proactively take action for health. Other screening motivations included family history of cancer, perceived symptoms of CRC, and awareness of elevated CRC rates among the Alaska Native population. Participants also reported feeling that colonoscopy was a more reliable test than other options, with greater accuracy and effectiveness at detecting polyps or early cancer.

Respondents were also asked whether they were aware of the mt-sDNA test and what they thought about using that instead of colonoscopy for CRC screening. A majority of people (72%) reported that they were unaware of the mt-sDNA option. Of the 28% who were aware of the mt-sDNA option, slightly over half (57%) were from high-intensity outreach communities, and less than half (43%) were from the medium-intensity communities who had received the mailed health education materials. Those aware of mt-sDNA were asked about potential interest in its future use. Responses aligned with initial reasons for choosing colonoscopy, including concerns about mt-sDNA novelty and test completion logistics, including a lack of running water for sanitation during home stool collection. However, respondents also said that healthcare access challenges, such as the lack of a medical escort and dislike of the colonoscopy bowel preparation process, might make them more interested in mt-sDNA for future screenings.

## 4. Discussion

Our findings align with previous research that highlights the mt-sDNA test’s ease of use, including minimal travel time, minimal disruption of daily activities, and less preparation time, as well as potential complications, invasiveness, and embarrassment associated with colonoscopy as major factors influencing screening test choice [22]. Those who chose mt-sDNA in our study noted specific colonoscopy concerns including fear, pain and discomfort, embarrassment, and test safety as factors in their test choice decision-making. Participants who chose colonoscopy for screening emphasized its reliability and accuracy and that it was performed by medical professionals, but noted the requirement for a medical escort and dislike of the bowel prep as reasons they might consider mt-sDNA in the future. This accords with other research that reported that only recommending colonoscopy can lower adherence to CRC screening, especially among racial/ethnic minorities, and that offering stool-based screening, such as mt-sDNA, or a choice between stool-based screening or colonoscopy, may enhance adherence to CRC screening [23].

This study also highlights the value that people place on their time when selecting a CRC screening method, as many participants reported preferring mt-sDNA due to the inconvenience of traveling for a colonoscopy and long wait times for appointments and limited endoscopy clinics, which were exacerbated by the COVID-19 pandemic occurring during the study intervention period. One surprising finding was that despite receiving the same outreach and test options, the majority of people who chose colonoscopy reported being unaware of the mt-sDNA option. There was a longer period between initial outreach and test completion among those who chose colonoscopy, which may have contributed to not remembering the other screening test option initially offered.

Many participants who chose mt-sDNA reported cost of travel and medical coverage as reasons for their decision. While both screening options were provided at no cost in the study, patients often bear the financial responsibility for travel, their escort, and the procedure if not covered by insurance. A recent national survey found that uninsured participants prefer stool-based testing options over colonoscopy, reflecting cost-related barriers [23]. This sentiment was echoed by colonoscopy respondents in our survey, who noted that they might choose mt-sDNA in the future if they lacked the required medical escort for travel and care. In addition to the health care access concerns, participants also shared they had personal logistical constraints, including work commitments and childcare responsibilities, which influenced their choice of using mt-sDNA over colonoscopy. Similarly, a recent survey of CRC screening-eligible individuals found that a lack of time for screening due to family responsibilities or having to take off work were significant barriers to receiving screening and led them to prefer less invasive alternatives to colonoscopy [24]. A number of respondents also noted that the telephone and mailed outreach from the study patient navigator influenced their decision to complete screening, which aligns with a New Hampshire CRC screening program evaluation which found that people in a patient navigation intervention group were 11.2 times more likely to complete colonoscopy than control patients not receiving navigation [25]. Integrating patient navigation more fully in rural Alaska Native communities could be a beneficial strategy for cancer screening and prevention within the Alaska Tribal Health System.

There were some study limitations. First, the survey sample was only among Alaska Native people in one region in Alaska. Therefore, study results may not be generalizable to Alaska Native peoples living in other parts of the state, or to American Indian populations in other areas of the U.S. Secondly, a higher proportion of women than men participated in the survey compared with non-responders, although the age group distributions and test outcomes were similar among respondents and non-respondents. Participants may not have fully understood the benefits and disadvantages of each screening test, so test choice and experiences might have differed with further knowledge. Additionally, other cultural factors beyond those brought forth by this group of individuals may exist in other Alaska Native populations which could influence participant behaviors, perceptions and responses, limiting the generalizability of these findings across diverse populations. Furthermore, the intervention trial occurred during the COVID-19 pandemic, which affected the timely availability of colonoscopy. The longer interval between outreach and testing may have contributed to the smaller proportion of colonoscopy survey respondents, and also may have influenced CRC screening test decisions and preferences.

The results of this survey provide valuable insights into preferences between mt-sDNA and colonoscopy, with implications for other noninvasive CRC screening methods, especially in tribal and rural/remote populations. These findings also underscore the complex factors influencing CRC screening among Alaska Native people. Limited access to medical facilities, financial burdens, and time commitments, along with personal factors, have a substantial bearing on the screening decision-making process. Recognizing and addressing these multifaceted influences and offering screening test options can help CRC screening programs and healthcare organizations better meet community member needs and preferences, ultimately improving screening rates and colorectal health outcomes.

## Figures and Tables

**Table 1:** Demographic information of colonoscopy and mt-sDNA survey responders and non-responders.

	mt-sDNA Responders		mt-sDNA Non-Responders		Colonoscopy Responders		Colonoscopy Non-Responders	
	n	%	n	%	n	%	n	%
Intervention Arm (Total) Medium intensity High intensity	113 44 69	58.9 38.9 61.1	79 31 48	41.1 39.2 60.8	51 20 31	39.2 39.2 60.8	79 37 42	60.8 46.8 53.2
Sex								
Male	62	54.9	53	67.1	20	39.2	41	51.9
Female	51	45.1	26	32.9	31	60.8	38	48.1
Age								
45–60 years	78	69.1	59	74.7	40	78.4	62	78.5
61–75 years	35	30.9	20	25.3	11	21.6	17	21.5

**Table 2:** Colonoscopy and mt-sDNA patient interview themes, subthemes, and supporting quotes, 2024.

Themes and Subthemes	Supporting Quotes
MT-sDNA	
Personal logistical concerns	
Travel inconvenience	“It just was more efficient. I didn’t have to travel”.
Work time constraints	“I wasn’t sure if I’d have enough [paid time off] to keep traveling”.
Personal time constraints	“Because, you know how spring is, very busy. Busy days, subsistence, all that stuff”.
Childcare	“Colonguard [mt-sDNA] I could do at home without traveling and having the hassle of looking for a sitter for my 5-year-old”.
Physical limitations	“Cause I can’t pretty much get anywhere…because of my limitations and movement, I have to be in a wheelchair”.
Ease/convenience Better than alternative	“It was offered, it’s more convenient and I don’t live near a medical facility”. “Home test kit, it seemed better for me. Less invasive I guess than colonoscopy”.
Negative perceptions of colonoscopy	
Test invasiveness	“Because I don’t like those probes going into my behind”.
Safety concerns	“I knew some patients who had internal bleeding after having a colonoscopy, so I wanted to do the at-home testing kit”.
Embarrassment	“It’s embarrassing you know, like if I have to go to the bathroom, I can’t hold it”.
Health care access	
Cost of travel	“Airfare was really expensive; I could just stay home to complete the screening”.
Medical coverage No medical escort COVID-19 pandemic	“Cause I don’t have Medicaid and I cannot pay for myself to go [to the regional hub hospital] to get screened”. “I haven’t had any symptoms and easier than trying to find an escort…” “At the time, I didn’t want to go in and risk getting COVID”.
Challenges using mt-sDNA Collection difficulties Inclement weather	“It was hard for me, the catcher. So next time we’re just going to do the colonoscopy”. “I actually started when I had to wait until the weather cleared up from the blizzard last winter—first time getting that box. The third one finally turned in last week”.
COLONOSCOPY	
Screening recommended	“Because I received a letter in the mail and needed to get checked out…”
Preventative health behavior Taking action	“Because it was recommended for my age and to catch any kind of early cancer”. “They asked if I wanted one, so I decided to because I have a brother and cousins that had problems with their intestines”.
Screening motivation Family history of cancer Higher risk among Alaska Native people	“Because there is a history of cancer in my family, so I wanted to get myself checked out”. “Because there were some ads saying a lot of Alaska Natives were getting a lot of colon cancer and that it is undetectable unless you have a colonoscopy”.
More reliable test	“Better screening then a stool sample. Because it is more reliable”.
Thoughts on using mt-sDNA instead of colonoscopy	
Concern about accuracy/novelty Test logistics No medical escort Dislike of colonoscopy prep	“I’m not too sure about that. It might be too new”. “We don’t have running water in the village so it would be hard”. “Maybe the next screen I will try it or if I have no escort, I would do the home screen”. “I would be interested since you wouldn’t have to drink the bottles of liquid and run to the bathroom”.

## Data Availability

Data are not available due to tribal research data restrictions. Researchers interested in these data may contact the corresponding author for more information on the process for requesting access to these tribal data.
